# Attracting Common Carp to a bait site with food reveals strong positive relationships between fish density, feeding activity, environmental DNA, and sex pheromone release that could be used in invasive fish management

**DOI:** 10.1002/ece3.4169

**Published:** 2018-06-11

**Authors:** Ratna Ghosal, Jessica J. Eichmiller, Bruce A. Witthuhn, Peter W. Sorensen

**Affiliations:** ^1^ Department of Fisheries, Wildlife and Conservation Biology University of Minnesota Saint Paul Minnesota; ^2^ Center for Mass Spectrometry and Proteomics University of Minnesota Saint Paul Minnesota; ^3^ Present address: Biological and Life Sciences School of Arts and Sciences Ahmedabad University, Central Campus Navrangpura, Ahmedabad Gujarat India; ^4^ Present address: Alexandria Technical and community College Alexandria Minnesota USA

**Keywords:** Carp, eDNA, feeding, invasive, pheromone, prostaglandin F_2α_

## Abstract

Measurement of environmental DNA (eDNA) is becoming a common technique to survey for rare and invasive fish due to its sensitivity and specificity. However, its utility is limited by an incomplete understanding of factors governing its sources and fates. Failure to detect eDNA is especially difficult to interpret so surveillance techniques often collect large numbers of samples across broad regions. If, however, fish could be reliably attracted to a single location where their eDNA could be easily measured that would be useful. We conducted a proof‐of‐concept study of this idea using invasive Common Carp. We monitored the distribution of radio‐tagged Carp and their eDNA across a 67 ha lake focusing at the bait site while a pheromone (Prostaglandin F_2α_; PGF_2α_) was also measured to determine their reproductive condition. Prior to baiting, Carp were patchily distributed and while eDNA was occasionally detectable, it was patchy and only loosely associated with moderately dense groups of fish. Further, neither Carp, nor their eDNA were consistently measurable at the bait site and surrounding region, and the pheromone was not measurable at all. However, once baiting commenced, Carp started visiting the bait site and feeding, especially at night, where eDNA levels increased 500‐fold as fish densities doubled and PGF_2α_ became detectable. Fish presence, eDNA and pheromone concentrations peaked at night after 6 days, strongly suggesting feeding activity was the main driver. While the presence of eDNA precisely coincided with this aggregation, levels had dropped dramatically within 5 m. PGF_2α_ levels dropped less rapidly and demonstrated the presence of live mature fish. We suggest that food could be used to train fish to come to locations where they otherwise are too scarce to be reliably measured, increasing their eDNA release, making them measurable, and their reproductive condition also discernable by measuring pheromones.

## 1 INTRODUCTION

The ability to detect a species and to ascertain its reproductive condition is a central theme in the prevention and management of invasive and rare species. However, no single approach has so far been able to accomplish this goal for fish (Evans & Lamberti, 2018; Harvey, Qureshi, & MacIsaac, 2009; Jerde, Mahon, Chadderton, & Lodge, 2011). Nevertheless, surveying for fish using the DNA they release (environmental DNA or “eDNA”) has become a method of choice because of the sensitivity and specificity of the technique, and the ease with which water can be collected (Bylemans et al., 2017; Ficetola, Miaud, Pompanon, & Taberlet, 2008; Jerde et al., 2011; Lacoursière‐Roussel, Rosabal, & Bernatchez, 2016; Minamoto et al., 2017; Taberlet, Coissac, Hajibabaei, & Rieseberg, 2012; Takahara, Minamoto, Yamanaka, Doi, & Kawabata, 2012; Thomsen & Willerslev, 2015). However, the utility of measuring eDNA is presently limited by the fact that many basic tenants of the ecology of eDNA are not well understood (Barnes & Turner, 2016; Evans & Lamberti, 2018). In particular, little is understood about either the origins of eDNA or factors that drive its release. Additionally, while our understanding of eDNA degradation, dilution and binding has expanded in recent years (Barnes & Turner, 2016; Evans & Lamberti, 2018; Turner et al., 2014), exactly how these factors interact to determine the distribution and fate of eDNA released by free‐ranging fish in natural waters remains unresolved. Finally, eDNA does not provide any information on whether fish are alive. These unknowns greatly complicate the practical value of eDNA measurement for both ecologists and fisheries managers, especially when fish distributions are patchy, or fish are rare, and nondetection rates are high, as is often the case for newly invading species (Eichmiller, Bajer, & Sorensen, 2014; Hinlo, Furlan, Suitor, & Gleeson, 2017; Takahara et al., 2012). The Bigheaded (Asian) Carps, *Hypophthalmichthys* spp., a pair of Chinese river fish moving up the Mississippi River, exemplify this as their presence is presently monitored using large sampling grids to overcome high nondetection rates that are difficult to interpret (ACRCC 2017). Another example is the Common Carp, *Cyprinus carpio* (hereafter “Carp”), which is managed across much of the world for which accurate information on rare individuals found at invasion fronts is difficult to acquire and interpret (Eichmiller et al., 2014; Sorensen & Bajer, 2011). If the distribution of Carp and other fish could be altered in a predictable manner to bring otherwise uncommon fish to a single location for sampling, and the biological significance of the elevated concentrations of eDNA they might release were understood, the potential value of eDNA measurement as a fisheries surveillance strategy would be greatly enhanced.

Food‐finding activity and feeding is of special importance to fish ecology and distribution, and likely eDNA release for several reasons. First, evidence from a laboratory study of Asian Carp suggests that feeding increases eDNA release rates (Klymus, Richter, Chapman, & Paukert, 2015). Second, wild fishes are typically food limited and food distribution (and its addition) can attract fish causing them to aggregate (Bajer, Lim, Travaline, Miller, & Sorensen, 2010; Ferno, Huse, Jackobsen, & Kristiansen, 2006; Ryer & Olla, 1995; Schmidt, Reis‐Filho, Harvey, & Girrizo, 2017), likely enhancing eDNA concentrations. Third, many fish have an inherent ability to locate and remember food patches, an understanding of which might be used by fishery biologists to both predict and perhaps create aggregations, which might then be measured as an enhanced index of fish presence using eDNA (Bajer et al., 2010; Broglio, Rodriguez, & Salas, 2003; Hughes & Blight, 2000; Odling‐Smee & Braithwaite, 2003; Vargas, López, Salas, & Thinus‐Blanc, 2004). Indeed, many fish, including the Common Carp and Bigheaded Carps, can be trained to come to particular locations in lakes using food, and this trait has proven to be exploitable for removal in areas they are otherwise hard to measure or catch (Bajer et al., 2010; P. W. Sorensen, unpublished results; Robin Calfee, USGS, Columbia Environmental Research Center, MO, USA). Interestingly, feeding activity in many fish, including the Carps follows circadian patterns, which might also influence eDNA distribution but this too has not yet been considered (Bajer et al., 2010; Helfman, 1986). Of course, fishers also employ an understanding of fish behavioral ecology to locate and catch fish (Jones, 1992); however, to our knowledge, basic ecological tenants that determine fish distribution have not yet been tested to determine whether they could be paired with eDNA measurement to enhance its value to fishery managers.

While eDNA measurement is proving to have value to managers, it nevertheless is limited because it does not, and cannot, provide information on fish gender, reproductive condition, or health; indeed, dead fish also shed eDNA (Barnes & Turner, 2016). However, pheromones, chemicals which fish release to convey taxon‐specific information (Sorensen & Wisenden, 2015) naturally provide this information about fish. These cues, which fish detect with their olfactory sense, are known from laboratory studies to be released in large quantities, and are typically both gender and life‐stage specific, and could be measured by field biologists although this has not yet been systematically examined (Sorensen & Johnson, 2016). Further, they are especially well understood in the family cyprinidae and the Carps in particular (Sorensen & Stacey, 2004; Stacey & Sorensen, 2009). Prostaglandin F_2α_ (PGF_2α_), a pheromone released by mature female Common Carp and their relatives (Stacey & Sorensen, 2009), as well many other species (as parts of mixtures), is especially interesting because it has proven to be measurable using mass spectrometry (MS) (Lim & Sorensen, 2011, 2012). However, PGF_2α_ has not yet been systematically measured in natural waters and its likely relationship to the distribution and abundance of wild fish as well as the distribution of their eDNA is also presently unknown.

In this study, we tested the possibility that Common Carp, a relatively typical and invasive cyprinid (Sorensen & Bajer, 2011), might be reliably and easily induced to aggregate using bait at a specific location so that its presence and reproductive condition could be efficiently determined using both eDNA and PGF_2α_. We were especially interested in testing this possibility in areas where Carp densities are low and difficult to measure, and whether feeding might predictably enhance eDNA release rates. The possibility that a sex pheromone concentration might also be measurable and correlate with fish density and gender was of special interest, as was well as how quickly these cues dissipate in natural waters. Finally, the precise relationship between the presence–absence, distribution, and abundance of Carp and their eDNA was of interest because it could inform fisheries management. We chose the Common Carp as our model for our proof‐of‐concept study because it shares many attributes with many thousands of fishes including the Asian Carps; it is highly mobile, social, omnivorous, consumes large quantities of food, spawns seasonally, and often lives to over 50 years of age (Ghosal, Xiong, & Sorensen, 2016; Kolar et al., 2005; Sorensen & Bajer, 2011).

## 2 MATERIALS AND METHODS

### 2.1 Experimental design

The study proceeded in six steps which included: (1) study site selection; (2) radio‐tagging Carp in the study lake, initial tracking, and selecting a site to bait; (3) establishing the baseline distribution and movement patterns of adult Carp while evaluating eDNA patterns across the lake, and eDNA and pheromone concentrations at a test site for future baiting; (4) describing the distribution of adult Carp and their eDNA while baiting and then evaluating eDNA and pheromone concentrations at the bait site on a 24‐hr cycle; (5) measuring pheromone release by Carp under laboratory conditions to confirm field measurements; and (6) laboratory analyses of eDNA and the pheromone, and statistical analysis.

### 2.2 Study site selection

**Figure 1:**
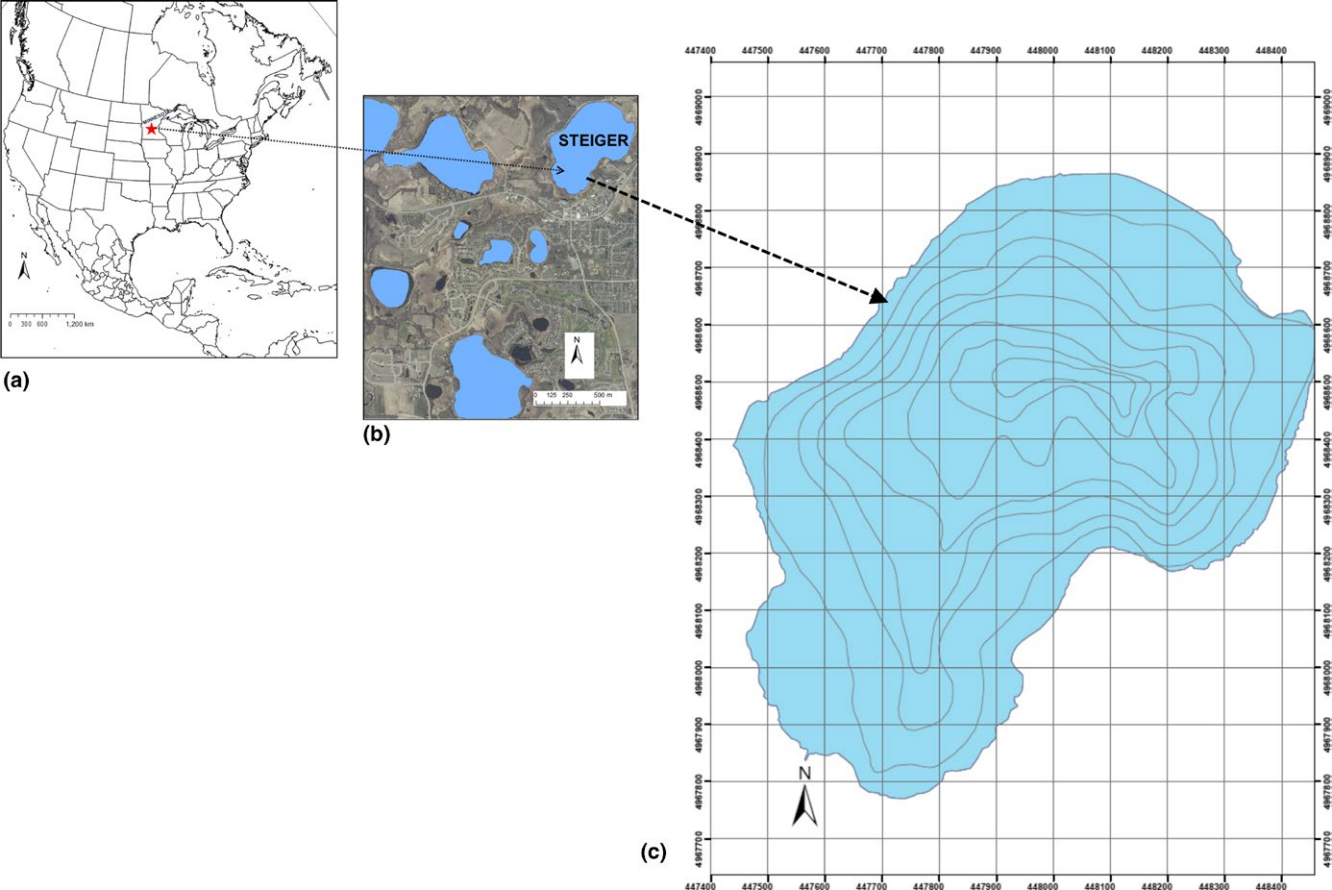
Map showing (a) North America, where 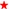 represents the state of Minnesota (Map Source: Google Maps); (b) Lake Steiger, the study site, and surrounding lakes (Map source: Google Maps); (c) Bathymetric and contour map of Lake Steiger (http://www.dnr.state.mn.us/lakefind/lake.html?id=10004500) depth contours are in five foot (~1.5 m) intervals

We examined several dozen lakes in Upper Mississippi River Basin for use as a possible site for this study and eventually chose Lake Steiger (44°52′5.0″N, 93°39′30.6″W, Figure 1) for several reasons. First, we knew from an ongoing mark–recapture study using electrofishing that it contained ~2,900 adult Common Carp (95% CI: 1,915–3,857) (Swanson, 2017), with a total biomass of about 150 kg/ha, a relatively modest, but not atypical density of Carp (Bajer & Sorensen, 2010, 2012), Second, the plant, animal, and fish communities in Lake Steiger (http://www.dnr.state.mn.us/lakefind/lake.html?id=10004500) are typical of other lakes in the Upper Mississippi River Basin. Third, aquatic habitat in the lake is also relatively typical and uniform with floating patches of cattail marsh and a heavy infestation of Eurasian milfoil. Finally, the lake has a surface area of 67 ha and an average depth of 4 m and no outlet or inlet, typical of many shallow lakes in this region.

### 2.3 Radio‐tagging carp and bait site selection

After selecting Lake Steiger, an electrofishing boat was used to systematically survey its fish populations after spawning ceased in July 2015. As adult Carp were encountered (no juveniles were seen), they were captured, anesthetized, and a radio‐tag inserted into their body cavities following established procedures (F1850, Advanced Telemetry Systems, MN, USA; Bajer & Sorensen, 2012; Penne & Pierce, 2008), after which they were released back into the lake at their capture location. We tagged 30 adult Carp (12 males and 18 females). A previous study showed that this approach (“Judas fish”) can be used to accurately describe overall Carp distribution and abundance (Bajer, Chizinski, & Sorensen, 2011). We re‐located these Carp 3 weeks later from a small boat using radio‐telemetry (manual bi‐angulation, a technique with an accuracy of about 20 m; Bajer & Sorensen, 2010) to confirm their health (all survived) and to pick a site that lacked Carp that could be used as a bait site. We selected the northern corner of the lake as a bait site. This location had few plants and was 5 m deep. An automated stationary radio receiver and data logger (Advanced Telemetry Systems, Isanti, MN, USA), which scanned all radio frequencies 10 times/h, was placed on shore at this site. Tests showed that it could detect the presence of radio‐tagged Carp within a distance of 150 m.

### 2.4 Establishing baseline carp distribution, eDNA, and pheromone levels

**Figure 2:**
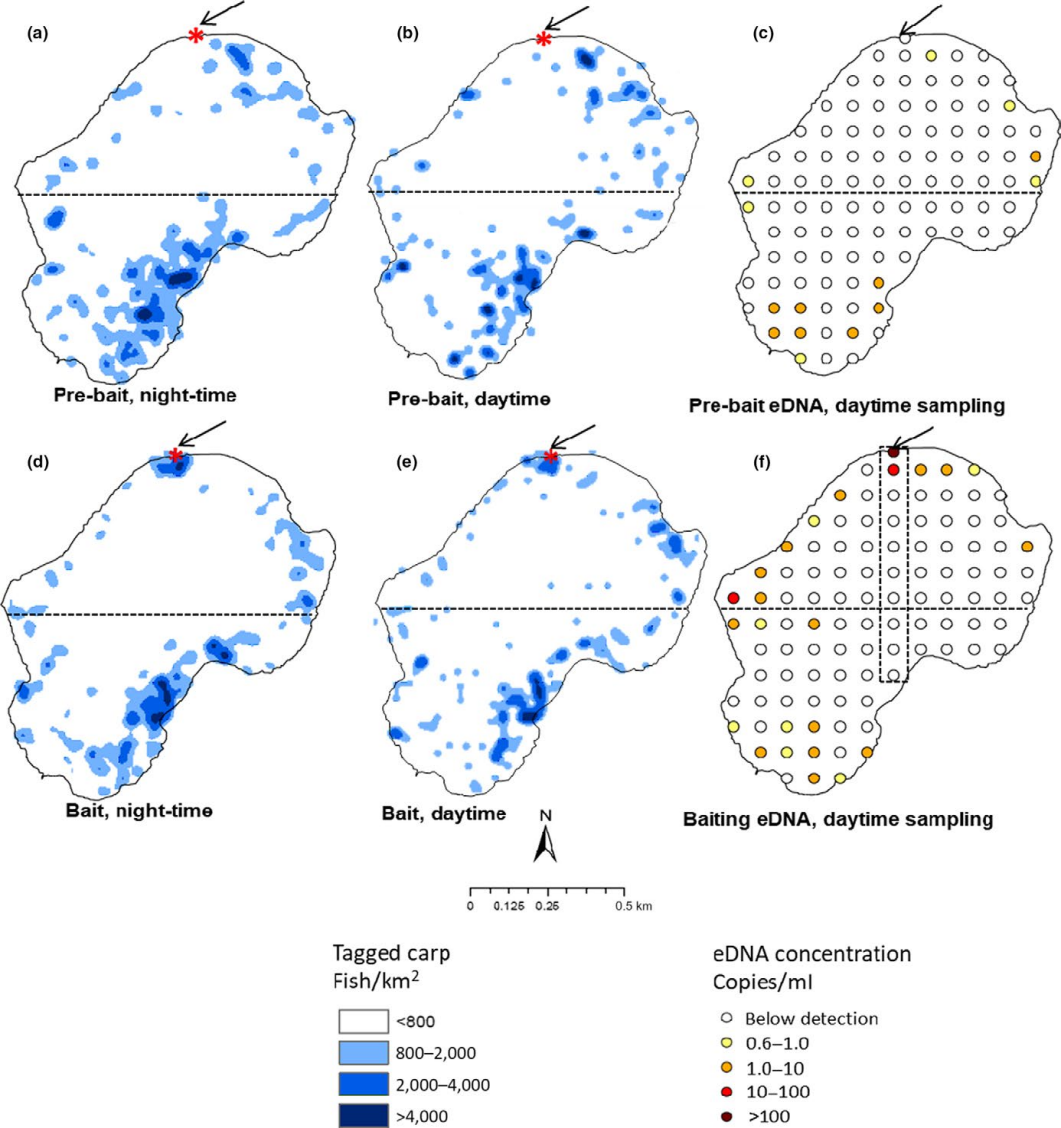
Map of Lake Steiger showing the distribution of radio‐tagged Carp and eDNA levels during the prebaiting and baiting phases. Panels (a, d) are kernel plots showing the distribution of radio‐tagged Carp during the nighttime (averaged over 7 nights) with panels (b, e) showing daytime values (averaged over 7 days) for the prebaiting and baiting phases. Panels (c) and (f) show the eDNA levels measured at each sample site the morning after the prebaiting and the baiting phases. The asterisk (*) and arrow identifies the bait site. The dotted line shows the northern and southern halves of the lake while the dotted rectangular box represents the sampling transect used at the end of the experiment

After selecting the bait site, we systematically started a 7‐day program (prebaiting phase) to determine the precise day–night distribution of all radio‐tagged Carp in the lake from a boat. Carp were located once each night (21:00–02:00 hours) and once each day (9:00–14:00 hours), and their positions calculated using LOAS^®^ 4.0 (Ecological Software Solutions, CA, USA) as our previous studies have suggested that they are nocturnal feeders (Bajer et al., 2010). During this time, empty mesh food bags were also placed as a control at the bait site which we lifted every 6‐hr while monitoring the presence of Carp within a distance of 150 m every hour using our automated receiver. Water samples for eDNA and pheromone analysis were also collected using 1‐L HDPE bottles at 03:00 and 15:00 hours each day at the bait site. At the end of this 7‐day period, we collected 100 water samples from across the lake at 08:00 hours (after daybreak) for eDNA analysis using a point‐intercept sampling design which had a grid size of 81 m (Figure 2).

### 2.5 Determining carp distribution, eDNA, and pheromone levels during baiting

Immediately after the 7‐day prebaiting phase, baiting commenced using cracked corn, following an established protocol (Bajer et al., 2010). Briefly, mesh bags were filled with dried cracked corn (25 kg/bag; Mills Fleet Farm, MN, USA), a favorite food of Carp (Bajer et al., 2010). These bait bags were lifted every 6 hr, weighed using a portable balance to determine food consumption, and re‐filled every 24 hr with fresh corn and placed back into the lake. This process continued for 7 days during which time the presence of radio‐tagged Carp within 150 m of the bait site was monitored every h using the automated receiver. Tracking cycles, telemetry procedures and water sampling methods were the same as during the baiting phase. In addition, at the conclusion of the baiting phase, we collected another set of 100 1‐L water samples from across the lake for eDNA analyses at 08:00 hours. Next, we collected water samples every 5 m along a north‐south transect extending 1,000 m from the bait to determine fine scale distribution of eDNA and the pheromone. Sediment samples were also sampled at the bait site at this time for eDNA by disturbing the bottom using an anchor for 5 min, and collecting sediment‐laden water in sample bottles. Finally, if floating fecal material was observed, it was noted and collected. All samples were immediately placed on ice in a cooler that had been cleaned with 10% bleach and transported back to the laboratory where they were stored at 4°C until they could be analyzed.

### 2.6 Confirming release rates of PGF_2α_

Because PGF_2α_ release rates by mature, nonspawning Carp had not been previously measured, we conducted a laboratory experiment so these values could be calculated and extrapolated to Carp in the lake. Three spermiated males (gonado‐somatic index [GSI; Mean ± *SD*] = 7.2 ± 0.3) and 3 vitellogenic females (GSI; Mean ± *SD* = 12.2 ± 0.4) weighing about 200 g were placed into plastic containers containing 10‐L of aerated 20°C well water for 1 hr following an established protocol (Lim & Sorensen, 2011). One litre water samples were then collected, spiked with 500 ng of deuterated PGF_2α_ (to serve as an internal standard), extracted, and analyzed following the protocol described in the pheromone analysis section.

### 2.7 eDNA analysis

Following established protocols, 1‐L water samples were first filtered through 47 mm, grade 934‐AH, glass microfiber filters (GE Healthcare Life Sciences, UK) using a polyphenylsulfone filter funnel (Pall Corporation, NY) (Eichmiller et al., 2014). Filters were folded in half and stored at −80°C in sterile Whirl‐Pak bags (Nasco, Fort Atkinson, WI, USA). Ten filtration controls, consisting of 1‐L of distilled water, were processed with each whole‐lake sampling event. Filtration equipment was decontaminated between samples using a 10‐min soak in 10% bleach, followed by liberal rinsing with distilled water. Extraction and quantitative PCR techniques were performed as previously described by Eichmiller, Best, and Sorensen (2016). Briefly, prior to extraction, filters containing water or sediment samples were sliced into 1 mm × 3 mm pieces using a sterile razor blade. Carp fecal samples were extracted directly from a 0.25‐g homogenized subsample of the fecal pellets. Filters and Carp feces were both extracted using the FastDNA Spin Kit (MP Biomedicals, Santa Ana, CA, USA). Samples were homogenized for 40 s on a speed setting of 6.0 using the FastPrep Instrument (MP Biomedicals, Santa Ana, CA) in 1 ml of extraction buffer CLS‐TC. A blank extraction tube was included with each set of samples extracted to serve as an extraction control. Potential PCR inhibitors were removed from eluted DNA after extraction using the OneStep PCR Inhibitor Removal Kit (Zymo Research Corp, Irvine, California). DNA extracts were stored at −80°C until qPCR analysis.

Quantitative PCR was performed as previously described (Eichmiller et al., 2016) using a qPCR marker specific for Common Carp that targets mtDNA cytochrome oxidase b gene. Triplicate negative PCR controls and copies of standards were included in each qPCR reaction plate, as previously described (Eichmiller et al., 2016). Each water sample had three analytical replicates (i.e., each extracted water sample was run in three different PCR reactions and results were averaged). To test for inhibition of amplification of Common Carp eDNA, DNA from 14 random samples from each whole‐lake sampling event that failed to amplify were also spiked with 30,000 copies of Common Carp standard DNA. The spiked samples were run, and cycle threshold (Cq) values were compared to expected values to ascertain the presence of inhibition. All extraction, filtration, and qPCR controls for eDNA showed no amplification for the Common Carp marker, indicating the absence of contamination during laboratory processing of samples. *R* ^2^ values for qPCR standard curves were greater than 0.985, and the average qPCR reaction efficiency was 89% (Supporting information: Table S1). Inhibition testing indicated no suppression of amplification; on average there was a 0.13 difference in Cq values between control and spiked samples. The amount of sediment extracted ranged from 8.0 to 178 mg.

### 2.8 Pheromone analysis

Deuterated PGF_2α_ (500 ng; Cayman Chemical, Ann Arbor, MI, USA) was added to each 1‐L water sample to serve as an internal standard, after which water samples were extracted by passing them through activated reverse‐phase C18 columns (Sep‐Pak, Waters Inc., Milford, MA, USA) following established techniques (Lim & Sorensen, 2012). Columns were eluted with 5 ml of methanol (Sigma‐Aldrich, St. Louis, MO, USA), and dried under a stream of nitrogen (see Fine & Sorensen, 2005). Quantitative analysis was then performed using LCMS/MS Selective Reaction Monitoring (SRM) and the presence of PGF_2α_ confirmed in 25 samples using Selective Ion Monitoring (SIM; see below).

Quantitative (SRM) analysis was performed by reconstituting the dried sample tubes in 1 ml of Buffer A (63% water, 37% Acetonotrile, 0.02% Formic acid), 10 μl of which was then injected onto an analytical Vydac C18 column (5 μm, 2.2 × 250) where they were subjected to a gradient of Buffer A (63% water, 37% Acetonitrile, 0.02% Formic acid) to Buffer B (50% acetonitrile 50% isopropanol) for 18 min at a flow rate of 400 μl/min (Supporting information: Table S2) into an Applied Biosystem 4000 iontrap mass spectrometer fitted with a turbo V electrospray source. Instrument settings were determined using the compound optimization mode with direct injection of the native and stable isotope compounds. Compounds with similar mass and product ions were distinguished by their retention times based on the retention times of the standard curve and the internal standards in the sample (Supporting information: Table S3). Data were analyzed using MultiQuant^™^ (Sciex, Framingham, MA, USA) providing the peak area ratio standard curve, from picomole to nanomole of PGF_2α_ with values of the unknowns fitted to the *y *= *mx *+ *b* determined by MultiQuant. Samples and standards were run in duplicate, and concentrations determined with the standard curve. Standard curves to determine the concentration of PGF_2α_ were obtained by performing a linear regression analysis on standard solutions of PGF_2α_ ranging from 177.3 to 1.38 nM using the ratio of standard area (PGF_2α_) to internal standard area (deuterated PGF_2α_). The range of detection of PGF_2α_ was between 177.33 and 11.08 nM using this method (Supporting information: Figure S1).

In addition to quantitative analysis, a subset of 25 samples was subjected to qualitative analysis of PGF_2α_ using selective ion monitoring (SIM) monitoring with a high mass accuracy Q Exactive to confirm PGF_2α_ identity. These samples were diluted with starting buffer (buffer A; 63% water, 37% acetonitrile with 0.02% formic acid). A DIonex Ultimate 3000 UHPLC system (Thermo Scientific) was used to perform reverse‐phase chromatography. The UHPLC system was fit with a Waters Corp. Aquity BEH C18, 1.7 μm particle size, 2.1 × 100 mm column. Samples were loaded using buffer A, and gradient elution was performed using 50% acetonitrile, 50% isopropyl alcohol as buffer B. The gradient profile consisted of: 2 min 100% buffer A; from 2 to 6 min to 20% B; from 6 to 6.5 min to 55% B; isocratic flow at 55% B from 6.5 to 10 min; from 10 to 11 min to 100% B; isocratic flow at 100% B from 11 to 13 min; from 13 to 13.5 to 100% buffer A; and isocratic flow at 100% buffer A from 13.5 to 16 min. The flow rate was 0.25 ml/min, and the column was maintained at 40°C. Mass information was collected using a Q Exactive (Thermo Scientific), quadrupole/orbitrap mass spectrometer. The instrument was operated in single ion‐monitor mode and negative polarity. Spray voltage was 3 kV, and capillary and probe temperatures were 320°C and 400°C, respectively. Resolution was set at 35,000, isolation width at 1.5 *m/z*, automatic gain control set at 5e5, and maximum ion collection time at 300 ms. Masses in the inclusion list were 353.2333 and 357.2589, and data were collected from 3.9 to 4.6 minutes. For fragmentation analysis, full scan resolution was set at 35,000 while the MS/MS scan resolution was 17,500. Normalized collision energy was set at 50.

### 2.9 Statistical analyses

Initial analyses examined Carp distribution across the entire lake, as well as that of eDNA, and possible relationships between them. Later, we examined these relationships and the pheromone levels in a more precise and quantitative way at the bait site. The overall distribution of Carp across the lake was first visualized by calculating the kernel density (search radius of  35 m, ~1 *SE* of Carp location; output cell size =  4.2 m) of Carp locations using the spatial analyst tool in ArcMap (10.2, ESRI, Redlands, CA, USA). We evaluated the northern and southern areas of the lake separately of each other to determine local movement and distribution patterns. The average number (averaged over 7 days for each phase) of radio‐tagged Carp in each half of the lake was calculated during the prebaiting and baiting phases. We focused on high‐density groups as low‐density groups were poorly defined. For both the prebait and baiting phases, we counted the number of high‐density groups (2,000–4,000 fish/km^2^) using kernel plots. All the connected high‐density kernels were considered to be a group, irrespective of size. We also counted the number of sample sites which had both detectable eDNA and measurable densities of Carp.

To evaluate the abundance of Carp near the bait site, we used the automated data logger to calculate the average number of individual radio‐tagged Carp detected per hour (daytime period: 06:00–18:00 hours and nighttime period: 18:00–06:00 hours) as well as the average of total detections per h within 150 m of the bait site. We also calculated average eDNA and pheromone concentrations at the bait site. First, we tested all the data for normality (Shapiro‐Wilk test) as well as homogeneity of variance (Levene’s test). Next, because the data followed a normal distribution, we performed separate two‐way ANOVAs (Graph Pad Prism, version 5.0) with post hoc Bonferroni’s tests for each variable (i.e., the average number of individual radio‐tagged Carp per h and average of total detections of tagged Carp per h, copies of eDNA, and concentration of PGF_2α_) during the prebaiting and the baiting phases (independently of each other), to evaluate the effects of time‐of‐day (i.e., day or night), and time (i.e., day 1–7) as well as possible interactions at the bait site. If no differences (*p* = 0.05) were found during the prebait phase, these data were then combined to serve as day 0 (control) for the baiting phase analysis. Using this procedure, we simplified the analysis and avoided the unnecessary use of very complex, difficult to interpret three‐way ANOVAs (Bolker, 2008). If two‐way ANOVAs of baiting phase data were later found significant, we followed them up with separate one‐way ANOVAs with Tukey’s post hoc tests for each data set to identify the specific time points (day one to day seven) where values changed within a particular time period (day or night). This particular set of analyses was adopted after carefully considering alternatives including repeated‐measures ANOVA which was deemed inappropriate because different Carp visited the bait site each night so the data were independent (personal communication, Dr. Aaron Rendahl, University of Minnesota Institute for Research on Statistics and Applications, Minneapolis, MN, USA).

Finally, bait consumption was plotted without additional analysis because it reflected single summed measurements. Pheromone and eDNA concentrations along the sampling transect from the bait site were also plotted without formal analysis for the same reason. Correlations between eDNA concentration, Carp number, and sex pheromone concentration were calculated across time for both day and nighttime scenarios at the bait sites. Food consumption was simply plotted because these data did not lend themselves to statistical analysis because they were one‐time measurements.

## 3 RESULTS

### 3.1 The overall distribution of Carp and eDNA across the entire lake before and during baiting

The overall distribution of Carp was patchy and uneven during both the prebaiting and baiting periods across the lake. Carp distribution was relatively constant during the prebaiting period with individuals moving ~153 ± 52 m (mean ± *SD*) between day and night periods (Supporting information: Figure S2A). On average, 10 of the 30 radio‐tagged Carp were found in the northern half of the lake during the entire prebaiting phase, creating (when averaged), ~7 small discernable groups during the day and three during the night. Our one‐time sampling measured eDNA at low levels (<1 copy/ml) at 5/54 lake sampling sites of which two sites coincided with the two larger high‐density groups of Carp described both during the day and night (Figure 2). A similar pattern was noted in the southern half of the lake where most Carp were found along the southeast shore as 12 moderate‐sized high‐density daytime groups. Their eDNA was only measurable at 9/47 lake sample sites (>0.6 copy/ml), three of which co‐localized with robust groups of Carp. No eDNA was measured at the bait site by this particular survey.

The overall distribution of Carp shifted slightly after baiting commenced, and fish density was much higher at the bait site during baiting relative to prebaiting phase (Figure 2). In addition to the new bait site group, there were, on average, four small high‐density groups of Carp in the northern half of the lake during the day and three at night. eDNA detection rates (and levels) nevertheless remained sporadic. The number of sample sites with detectable eDNA in the northern half of the lake increased to 12 from 5 by day seven (Figure 2). Meanwhile, the southern half of the lake appeared little changed; it had, on average, eight high‐density groups of Carp during the day, and nine at night, very similar to that seen during the prebaiting phase. The number of sample sites with detectable eDNA in the southern half of the lake was little changed at 12, three of which coincided with high‐density groups. Calculations showed that the Carp detected at the baiting site had travelled an average distance of 357 ± 112 m to get to the bait site (Supporting information: Figure S2).

### 3.2 Carp, eDNA, and pheromone levels at the bait site both before, and during baiting

**Figure 3:**
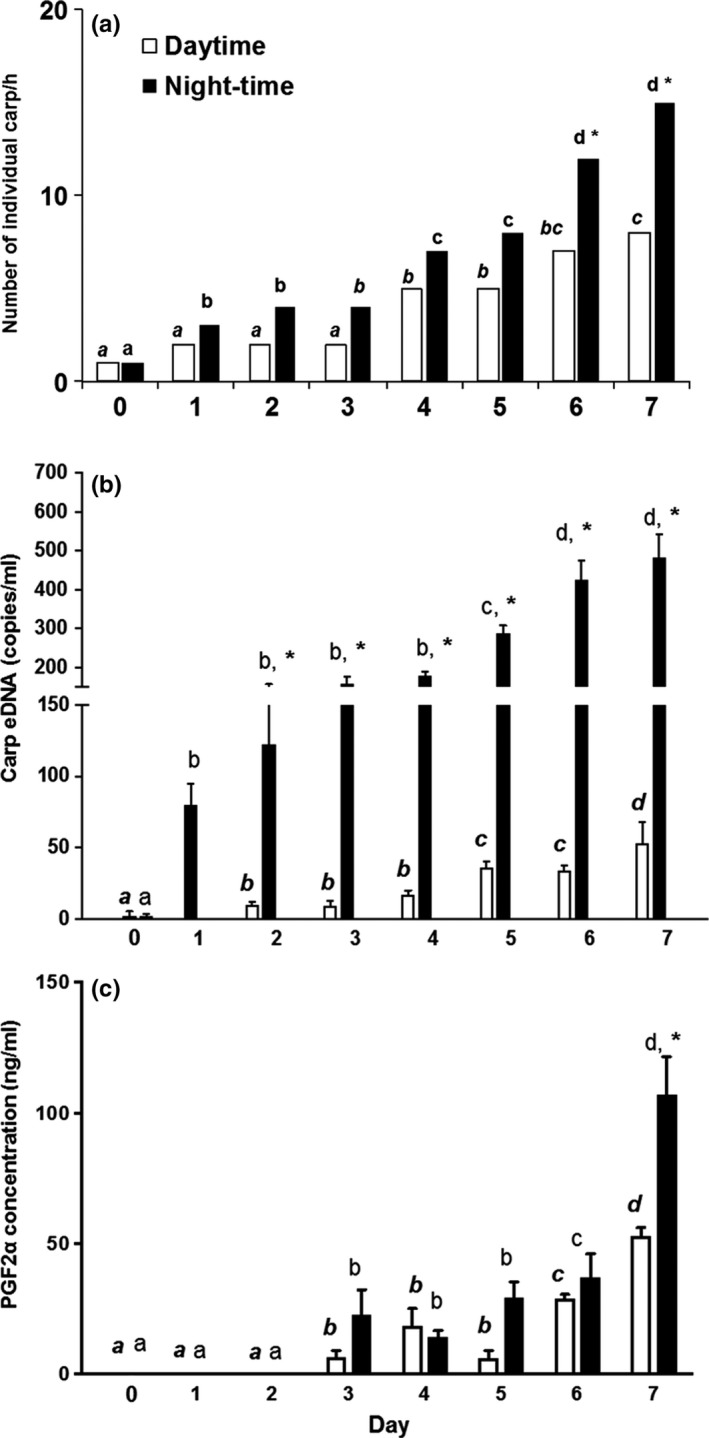
Average number of individual radio‐tagged Carp/hr, eDNA and PGF_2α_ concentrations at the bait site during prebaiting and baiting phases. (a) Number (*n* = 12) of individual radio‐tagged Carp/hr within 150 m of the stationary receiver; (b) eDNA concentration (copies/ml, mean ± *SD*,* n* = 3); (c) Prostaglandin (PGF_2α_) concentration (ng/ml, mean ± *SD*,* n* = 3). Day 0 is the average of prebaiting values in all three cases; * indicates days when nighttime measurements were different than daytime. Letters compare values within daytime periods (italicized) and nighttime (not italicized); all values are compared with their matched starting values with different letters indicating differences between them (*p* < 0.05)

Analyses showed that the number of individual Carp detected at the bait site was low (about 3/h) and constant during the prebait period with no daynight differences (Figure 3a) (two‐way ANOVA, *p* > 0.05) but increased soon after baiting started, with large, measureable increases at night (Figure 3a). By day seven, the number of individual Carp at the bait site was five times the number seen at the start of the experiment. A two‐way ANOVA demonstrated that the number of Carp increased with both time (day; *F*_7,7_ = 16.31, *p* < 0.05) and time‐of‐day (*F*_1,7_ = 17.21, *p* < 0.05) with no interaction (*p* > 0.05). Post hoc Bonferroni’s tests showed that the average number of Carp found at the bait site was greater during the night than during the day for all days, with differences (*p* < 0.05) at day six and seven (Figure 3a). Follow‐up tests found increases at day four and seven for the daytime period (one‐way ANOVA with Tukey’s post hoc tests, *F*_7,88_ = 13.24, *p* < 0.05), and at days one, four, and six for the nighttime period (One‐way ANOVA with Tukey’s post hoc tests, *F*_7,88_ = 6.34, *p* < 0.05) (Figure 3a).

**Figure 4:**
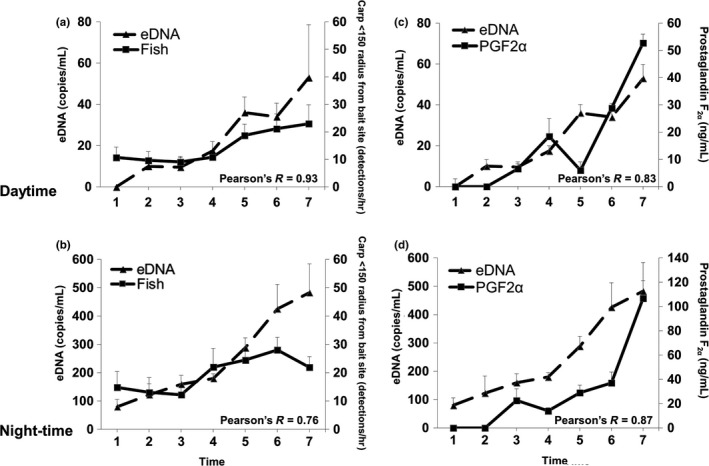
Plots showing the correlations between eDNA levels at the bait site with the total number of individual Carp across time (days since baiting started) as well as the concentrations of Prostaglandin F_2α_ (PGF_2α_) at the bait site during the daytime (a, c) and nighttime (b, d) of the baiting phase

Similarly, while eDNA concentrations at the bait site were extremely low, albeit constant (<0.5 copy/ml) prior to baiting (two‐way ANOVA, *p* > 0.05), very large increases in eDNA levels were measured immediately after baiting commenced, with nighttime values increasing ~50‐fold (Figure 3b). By day seven, these values had increased another order of magnitude and were about 10 times greater at night than day, reaching 500 copies/ml, a 500‐fold overall increase. Two‐way ANOVA confirmed these trends showing that eDNA levels increased with time (day 1–7, *F*_7,32_ = 32.93, *p* < 0.05) with day–night differences (*F*_1,32_ = 296.37, *p* < 0.05) and an interaction between the two (*F*_7,32_ = 21.5, *p* < 0.05). Except for day one, nighttime eDNA values were greater than their daytime values (two‐way ANOVA with post hoc Bonferroni’s tests, *p* < 0.05; Figure 3b). eDNA concentrations increased with time, with measureable increases at day five during both the daytime (one‐way ANOVA with Tukey’s post hoc tests, *F*_6,14_ = 4.26, *p* < 0.05) and nighttime (one‐way ANOVA with Tukey’s post hoc tests, *F*_6,14_ = 2.75, *p* < 0.05) (Figure 3b). The average number of eDNA copies per ml, and the number of individual Carp/hr at the bait site were positively correlated during both the daytime (Pearson’s correlation *R* = 0.93, *p* < 0.05) and nighttime periods (Pearson’s correlation *R* = 0.76, *p* < 0.05) (Figure 4), with the daytime relationship being the strongest. Feces were frequently seen at the bait site and the three fecal samples collected at the baiting site on the last day of baiting contained high concentrations of eDNA (1.6 × 10^7^, 1.8 × 10^8^, and 2.6 × 10^5^ copies/wet g).

Sex pheromone levels measured at the bait site showed similar but less dramatic trends than did eDNA, starting at low, undetectable values during the prebaiting period, but steadily increasing after three days of baiting with day–night differences evident at day seven (Figure 3c). The two‐way ANOVA showed significant time (day 1–7, *F*_7,16_ = 60.01, *p* < 0.05), day–night (*F*_1,16_ = 37.02, *p* < 0.05) as well as interaction effects (*F*_7,16_ = 9.45, *p* < 0.05). By day seven, the pheromone concentration during the night was higher than during the day (two‐way ANOVA with post hoc Bonferroni’s tests, *p* < 0.05; Figure 3c). Briefly, pheromone concentration increased over time with measurable increases at days three, six, and seven during both the daytime (one‐way ANOVA with Tukey’s post hoc tests, *F*_6,7_ = 10.61, *p* < 0.05) and nighttime sampling periods (one‐way ANOVA with Tukey’s post hoc tests, *F*_6,7_ = 9.18, *p* < 0.05). The mean concentration of PGF_2α_ at the bait site and the average number of eDNA copies/ml were also positively correlated (Pearson’s correlation *R* = 0.83, *p* < 0.05 during daytime, and Pearson’s correlation *R* = 0.87, *p* < 0.05 during the nighttime) (Figure 4c, d). Mature females were found to release 0.03 ng g^−1^ PGF_2α_ body wt hr^−1^ in the laboratory while males only released only one thirtieth of that, 0.001 ng g^−1^ body wt hr^−1^ PGF_2α._ Selective ion monitoring confirmed that we were definitively measuring PGF_2α_ in both lake and laboratory water (Supporting information: Figure S3).

**Figure 5:**
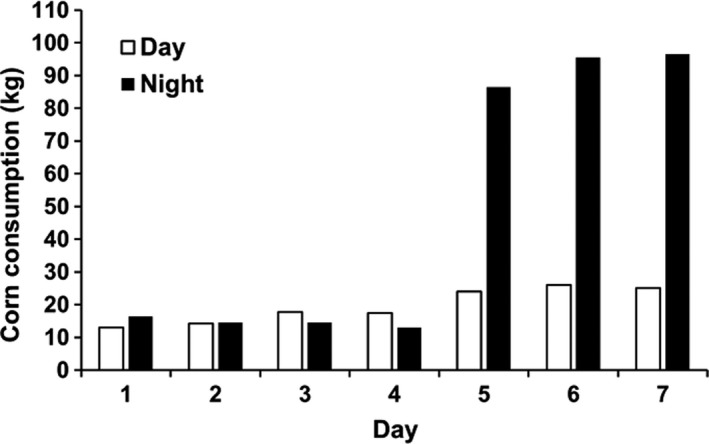
Total food consumption during the baiting phase for the daytime and nighttime periods

Food consumption at the bait site appeared to increase slowly, starting at ~10 kg/sampling period but doubling by day four, after which nighttime values increased nearly 10‐fold to over 100 kg/sampling period (Figure 5). This relationship may have been an underestimate because bait was often spilled when food bags were replaced, especially at night.

**Figure 6:**
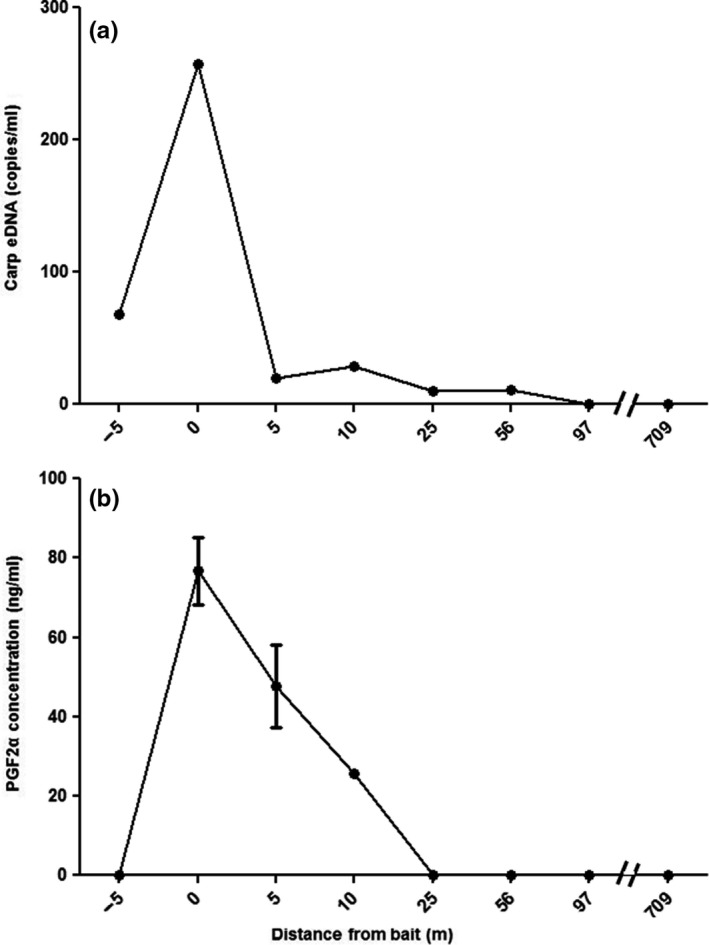
Concentrations of eDNA and PGF_2α_ along the north‐south sampling transect extending from the bait site showing: (a) eDNA (copies/ml) and (b) PGF_2α_ concentrations (ng/ml, mean ± *SD*,* n* = 3 assay replicates) at the end of the baiting phase. The baiting site is indicated by a “0”; the negative value is the shoreline

Analyses of eDNA and pheromone concentrations in the vicinity of the bait site at the conclusion of the experiment showed that while the concentration of eDNA at the bait site for was nearly 300 copies/ml, at a distance of five m it was only a tenth that value, a level it maintained for a distance of about 100 m before dropping below detection threshold (Figure 6a). A similar but less dramatic gradient was evident for the pheromone, which declined from a peak of ~80 ng/ml at the bait site to below detection at a distance of 25 m (Figure 6b).

## 4 DISCUSSION

This study makes several contributions to our understanding of the ecology of fish eDNA and how measurements of eDNA might be used as a management tool to detect and measure fish. First, and perhaps most importantly, we demonstrate that actively feeding Carp release approximately an order of magnitude more eDNA than fish feeding less actively. This relationship between fish behavior and eDNA release may well explain much of the variation commonly noted in eDNA measurements in lakes and rivers (where fish behavior is not typically noted) but could be exploited (as shown here) to make fish much easier to detect. Second, we show for the first time that we can systematically measure a teleost sex pheromone in a natural setting and that it can be used together with eDNA, to confirm the presence of living fish, and infer gender. Third, we confirm that a relatively common omnivorous invasive fish, the Common Carp, can easily and reliably be induced to aggregate at particular locations using food, where it can then be detected with great sensitivity and/or removed. Fourth, we show that nature and time of eDNA sampling schemes is important: eDNA presence best reflects fish presence when they were actively feeding and this occurs for Common Carp at night; clearly, eDNA surveillance schemes should account for the behavior of fishes. Additionally, we found that eDNA dissipated within just a few meters of the release site in natural waters, emphasizing the importance of understanding and accounting for fish density relationships and rapid eDNA decay in eDNA surveys. Similar to other studies (Eichmiller et al., 2014), we show once again that while eDNA accurately predicts the highest densities of fish, fine scale data must be taken in context because, as we also now show, eDNA dissipates very quickly. Because the Common Carp is a relatively unremarkable fish both ecologically and physiologically, these concepts should now be explored in other fishes.

From an ecological perspective, the most important finding of this study was that feeding activity naturally and quickly increased eDNA release by Carp, strongly suggesting feeding activity naturally drives very large increases in eDNA release. While the 500‐fold increase in eDNA concentration we measured was likely associated with a number of factors including increased fish biomass, it seems probable that feeding activity was the most important driver. Several lines of evidence point to this possibility. First, eDNA concentrations were 10 times greater at night than during the day when bait consumption was also 10 times greater. Second, Carp fish were only about twice as abundant at the bait site at night as during the day so Carp number (biomass) alone could not have been the main factor although likely it had a role. Third, total activity (detections) of Carp at night was no greater than during the day, suggesting fish swimming activity per se was not the cause (in fact, the typical Carp was likely less active when feeding at night; Supporting information: Figure S4). A previous laboratory study on Asian Carp showed that feeding was associated with increased eDNA release although it sampled across much longer time periods than we did and suggested feces, not feeding activity was responsible (Klymus et al., 2015). However, although feces clearly contributed to eDNA release in our study, the strong day–night pattern we observed cannot be explained by accumulating feces as feces were there night and day. Instead, it is likely that food processing and sorting behavior was the primary cause in our study. Common Carp are bottom feeders, which sort large quantities of sediment using a combination of their gill rakers, pharyngeal teeth, and a palatal organ awhile rooting in the bottom to find small food items (Bajer et al., 2010), a process that is likely associated with a release of cellular debris and mucus that could carry eDNA. The possibility that food processing drives eDNA is not precluded by previous results in the Carps as the Asian Carps, which are filter feeders, employ buccal pumping, a highly energetic process used to accumulate food from their gill rakers and accumulate it in mucous in their epibranchial organs (Hansen, Ghosal, Caprio, Claus, & Sorensen, 2014). Of course, feeding Carp must also be urinating as well as interacting physically (pushing each other, etc.), all of which have already been suggested to serve as sources of eDNA (Barnes & Turner, 2016; Klymus et al., 2015). Future studies should explore the specific relationship between feeding behavior as well as physiology and eDNA release in other Carps as well as other species of fish.

Our study may be the first to systematically measure a teleost fish pheromone in natural waters and adds a new surveillance tool to fisheries management as well as a new understanding of fish chemical communication and pheromone dispersion in natural environments. Using a new combination of SRM and Q exactive analyses coupled with a HPLC system, we were able to identify and accurately measure PGF_2α_ in induced aggregations of wild Carp to confirm that they were alive—an attribute eDNA cannot measure. Further, given that laboratory females released at least 30 times more PGF_2*a*_ than males, it seems highly likely that mature females were the primary source. Because spawning mature females even higher quantities of PGF_2α_ and a few of its metabolites, this technique could be expanded to determine precise reproductive state (Lim & Sorensen, 2012; Sorensen & Johnson, 2016). The PGF_2α_ release rates that we measured in mature female Carp were similar to those measured by Lim and Sorensen (2012) in mature Carp prior to spawning. Unlike previous studies on nesting Sea Lamprey, *Petromyzon marinus*, our study described a relationship between pheromone concentration and abundance in streams. It is interesting that the pheromone persisted much longer in lake water than eDNA suggesting that it has different chemical properties than eDNA which could be further exploited. Future work should determine decay and dilution rates of PGF_2α_ and its metabolites in open waters. The mass‐spectrometry method we employed was similar to that used for the Sea Lamprey (Fine & Sorensen, 2005; Stewart, Baker, & Cooney, 2011; Xi et al., 2011), and it could be improved. The combined use of sex pheromones and eDNA could be applied to measurement of the thousands of fish that also use prostaglandin‐based sex pheromones (Stacey, 2015; Stewart & Sorensen, 2015).

Our results align with previous findings that baiting can be used to predictably attract Carp to specific locations from broad, diffuse locations in lakes where they can also be easily measured or perhaps removed (Bajer et al., 2010; P. W. Sorensen, unpublished results). Both this study and Bajer et al. (2010) found that Common Carp took 3–4 days to arrive at a bait site in large numbers, strongly suggesting this it is a learned response. In both studies, nearly a third of each lake’s Carp came to the bait site (12/30 radio‐tags in Lake Steiger, perhaps reflecting about a thousand fish). However, while Bajer et al. (2010) attracted Carp from nearly 600 m away an across an entire lake, we were only able to attract fish from a distance of ~400 m, about half the lake. This difference might be attributable to different resource availability and home ranges in the two lakes and warrants study as it has bearing on how baiting can be used to survey and/or remove Carp. Indeed, we have started this work with Asian Carps which seem equally trainable to come to locations and also appear to release greater levels of eDNA when feeding (R. Ghosal & P. W. Sorensen, unpublished results). Likely the ability to attract fish with food is influenced by food availability and temperature (hunger), factors that warrant study.

The findings of our study have implications for how eDNA surveillance should be used in fisheries surveillance and management. Notably, we were able to bring Common Carp to a single location from nearly half a km away within a just few days and induce them to release levels of eDNA that exceeded those measured during the prebaiting phase, easily allowing us to positively confirm their presence in an area where they previously had been statistically undetectable (error bars for our measurements were below 0). This approach contrasts with a commonly used scheme used for Asian Carps that surveys using a quasi‐random grid based on surface area that typically employs well over a hundred points in most river reaches, does not overtly consider fish behavior or time‐of‐day (ACRCC 2017), and then follows up with capture surveys if eDNA is detected. We suggest that both the reliability of eDNA surveillance schemes could be increased using targeted baiting, perhaps with Judas fish to confirm efficacy. Increased, verifiable sensitivity, especially in low‐density areas, should also increase confidence in eDNA surveys and their use by management. The Laurentian Great Lakes are presently being surveyed for Asian Carp using eDNA and a shoreline baiting strategy work well there, just as it was in Lake Steiger. It might be also useful in the outlying, connected wetlands that Common Carp frequently move into to spawn, and where managers want to monitor Carp distribution and abundance but sampling is challenging (Dauphinais, Miller, Swanson, & Sorensen, 2018).

In addition to lakes, baiting might also be useful for eDNA surveys in the large rivers and floodplains where Asian Carps are presently of greatest concern. Flow need not necessarily be a serious complication in rivers; indeed, flow can create more detectable and larger eDNA plumes as has been proven to be the case for stream trout eDNA (Jane et al., 2014) and a Sea Lamprey sex pheromone (Xi et al., 2011). Food could be added from fixed baiting stations below dams, perhaps along with other cues such as sound to facilitate learning. Clearly, it will be important to identify and use the most attractive foods in optimal manners to motivate Asian Carp (Claus & Sorensen, 2017). Finally, although it seems likely that baiting could increase the sensitivity and ease of using eDNA for surveillance, its utility may ultimately be limited by uncontrollable ecological and behavioral factors such as hunger and competitiveness as noted for invasive crayfish trapping (Larson & Olden, 2016). Nevertheless, baiting has worked well, and without apparent bias, for video‐assisted fish censuses in the marine environment (Harvey, Cappo, Butler, Hall, & Kendrick, 2007). Studies of how Asian Carps might be baited in, and to, different places at different times of the year as part of a targeted eDNA surveillance scheme should be considered.

Our finding that Common Carp move, aggregate, and feed at night, and that this nocturnal activity triggers large releases of eDNA which then dissipate quickly also has important implications for understanding how eDNA surveillance data should be interpreted. Most importantly, it suggests that there may not always be a simple linear relationship between wild fish biomass and eDNA concentration because behavior is factor (Barnes & Turner, 2016). Other behaviors may have similar effects on eDNA, especially spawning (Erickson et al., 2016). Finally, while our finding that eDNA rapidly dissipated within just 5 m reinforces our previous results (Eichmiller et al., 2014) as well as numerous laboratory studies (Barnes & Turner, 2016; Eichmiller et al., 2016; Lance et al., 2017; Turner et al., 2014) which describe rapid decay and binding; further research is needed to precisely estimate degradation rates and the many factors that likely influence it in natural waters, especially in rivers.

Lastly, our study is only the second we know of to examine the precise relationship between the known distribution of free‐ranging fish and eDNA concentrations on a fine scale (i.e., a few m). Importantly, both studies (Eichmiller et al., 2014; the present study) find that although the distribution of eDNA can be patchy it does correlate well with very dense aggregations of Common Carp (but not smaller groups). Similar studies on fish distribution and eDNA levels in experimental outdoor ponds also report generally positive results (Takahara et al., 2013). Studies using tracked fishes and the eDNA and the pheromones they release in lotic environments might also prove insightful.

In conclusion, our approach of using food to attract wild fish to a location where their eDNA and sex pheromones can then be easily measured with great sensitivity, sheds new light on the ecological sources and fate of eDNA, and provides an efficient alternative to the large‐scale eDNA surveys presently being used by fisheries management programs (ACRCC 2017). We also clearly demonstrate how an understanding of fish ecology and behavior (e.g., nocturnal activity) can be exploited in the future eDNA studies. This work compliments similar studies which considered migratory behavior and eDNA monitoring, and point toward a need to consider fish behavior in eDNA measurements (Uchii, Doi, Yamanaka, & Minamoto, 2017). Because all fish species feed and release sex pheromones as well as eDNA, our integrated approach of using baiting and measuring both eDNA and sex pheromones may have wide applicability. Future research should target development of pheromone biomarkers in species of management concern that already have eDNA markers while developing a better understanding of fish ecology and behavior, and its relationship with eDNA.

## AUTHORS’ CONTRIBUTIONS

PWS conceived of the idea. RG designed the study along with JJE who provided specific input on eDNA. RG analyzed the pheromone data and wrote the first draft. RG and JJE conducted the fieldwork and sample collection. JJE was responsible for eDNA analysis. BW developed the mass‐spectrometry techniques. All parties edited the manuscript.

## CONFLICT OF INTEREST

None declared.

## DATA ACCESSIBILITY

eDNA, pheromone and radio‐tracking data available from the Dryad Digital Repository: https://doi.org/10.5061/dryad.8qf25s3
