# Supplementary material for: Attracting Common Carp to a bait site with food reveals strong positive relationships between fish density, feeding activity, environmental DNA, and sex pheromone release that could be used in invasive fish management
Source: Ecol Evol. 2018 Jun 11;8(13):6714–27. doi: 10.1002/ece3.4169 (PMC6137546; doi:10.1002/ece3.4169)
Supplement: Supplementary file 1 [file ECE3-8-6714-s001.doc]

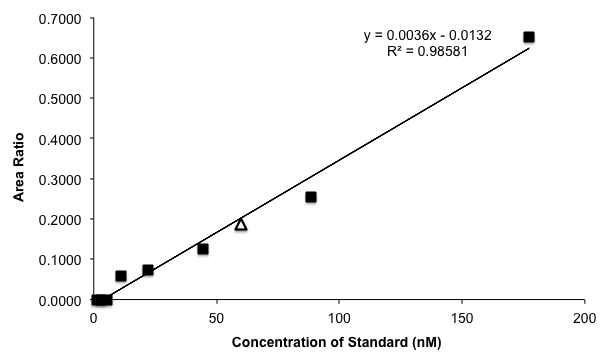
Fig. S1

Fig. S1: Standard curve for PGF_2α_ with a representative sample (Δ). X-axis represents nanomolar (nM) concentrations of standards and y-axis represents area ratio (area of standard concentration: area of internal standard, deuterated PGF_2α_).

Fig. S2A


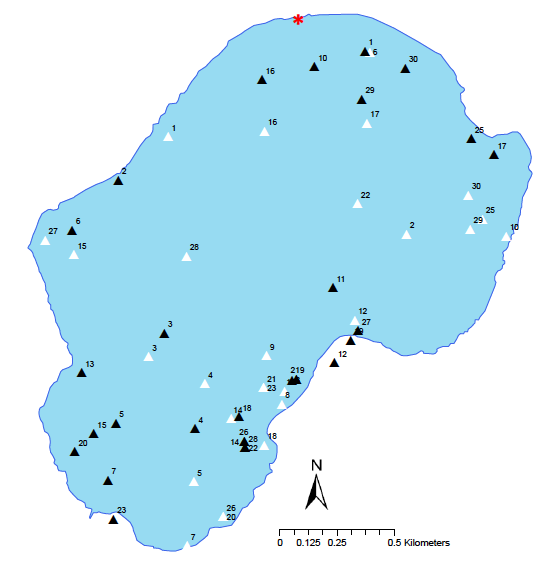


Fig. S2A: Day 7 locations of radio-tagged carp during the 24-hr cycle for pre-baiting phase, where ***** represents baiting site, represents daytime locations, and represents night-time locations of tagged carp. Numbers represent the unique tag code for each radio-tagged carp.

Fig. S2B

Fig. S2: Representative (Day 7) locations of radiotagged carp during the 24-hr cycle for pre-baiting (panel A) and baiting (panel B) phases, where ***** represents baiting site, represents daytime locations, and represents night-time locations of tagged carp. Numbers represent the unique tag code for each radiotagged carp.

Supplementary Fig. S3

Fig. S2B: Day 7 locations of radio-tagged carp during the 24-hr cycle for baiting phase, where ***** represents baiting site, represents daytime locations, and represents night-time locations of tagged carp. Numbers represent the unique tag code for each radio-tagged carp.

Fig S3

Fig. S3. SIM LCMS (negative) for the presence of M/Z of 353.23330 (lower panel) (PGF2α) and 357.25892 (upper panel) (deuterated PGF_2α_). The spectra were extracted using these masses with a threshold of 50 ppm.

Fig. S4


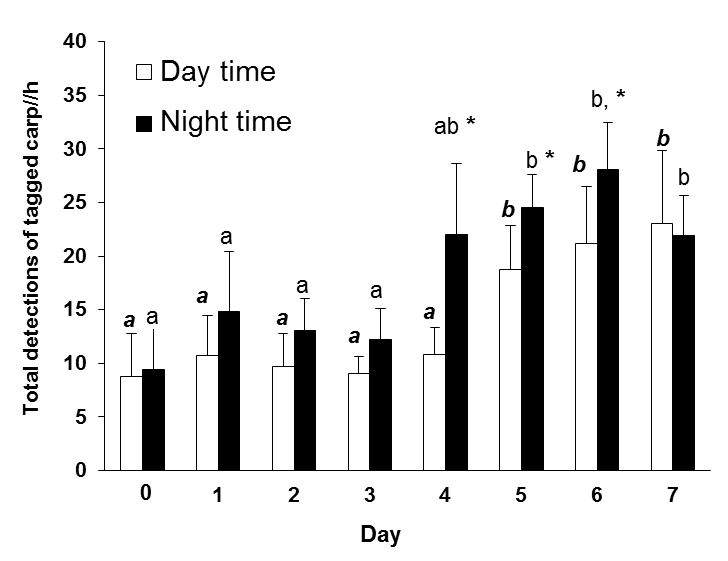


Fig. S4: Total number of detections of all radio-tagged carp per hour within <150 m of the stationary receiver at the bait site (Mean ± SD, n=12) (all detections are combined). * indicates the period during which the night-time measurements were higher than the daytime periods (Two-way ANOVA, P<0.05) while different letters indicate differences between values at different time points (day 1 to day 7, One-way ANOVA, P<0.05) within a particular time period (daytime or night-time). Comparisons between daytime values are italicized.

Table S1. Summary of calibration curve data across 13 qPCR runs.

| Parameter | Slope | Intercept | R^2^ |
| --- | --- | --- | --- |
| Mean ± SD*^a^* | –3.63 ± 0.15 | 39.86 ± 2.13 | 0.995 ± 0.003 |
| Minimum | –3.95 | 35.31 | 0.989 |
| Maximum | –3.42 | 42.83 | 0.999 |

*^a^*Standard deviation.

Table S2: Gradient profiles of buffers in SRM analysis

| Time | Flow Rate | Buffer A % | Buffer B % | |
| --- | --- | --- | --- | --- |
| 0 | 300 | 98.0 | 2 |  |
| 12 | 300 | 2.0 | 98.0 |  |
| 14 | 300 | 2.0 | 98.0 |  |
| 14.5 | 300 | 98.0 | 2 |  |
| 21 | 300 | 98.0 | 2 |  |
|  |  |  |  |  |

Table S3: Mass spectrometry profile for different prostaglandins spiked in control lake water sample

| **Prostaglandins** | **Q1 Mass** | **Transitions** | **Retention time** |
| --- | --- | --- | --- |
| PGE1 (Deuterated) | 357.2 | 321 | 4.03 |
|  |  | 277 |  |
|  |  | 239 |  |
| PGD1 (Deuterated) | 357.2 | 321 | 3.97 |
|  |  | 277 |  |
|  |  | 239 |  |
| PGF2α (Deuterated) | 357.2 | 197 | 3.51 |
|  |  | 169 |  |
| PGF2α | 353.2 | 193 | 3.51 |
|  |  | 165 |  |
| PGH1 | 353.2 | 317 | 3.84 and 7.89 |
|  |  | 273 |  |
|  |  | 235 |  |
| PGF2β | 353.2 | 309 | 2.92 |
|  |  | 291 |  |
| 11betaPGE1 | 353.2 | 335 | 4.07 |
| 13,14 dihyro 15Keto PGF2α | 353.2 | 165 | 6.7 |
|  |  | 193 |  |
| 15Keto PGF2α | 351 | 289 | 5.18 |
|  |  | 315 |  |
